# A High-Level Control Algorithm Based on sEMG Signalling for an Elbow Joint SMA Exoskeleton

**DOI:** 10.3390/s18082522

**Published:** 2018-08-02

**Authors:** Dorin Copaci, David Serrano, Luis Moreno, Dolores Blanco

**Affiliations:** Department of Systems Engineering and Automation, Carlos III University of Madrid, 28911 Leganés, Madrid, Spain; davserra@ing.uc3m.es (D.S.); moreno@ing.uc3m.es (L.M.); dblanco@ing.uc3m.es (D.B.)

**Keywords:** exoskeleton, electromyographic (EMG), control systems

## Abstract

A high-level control algorithm capable of generating position and torque references from surface electromyography signals (sEMG) was designed. It was applied to a shape memory alloy (SMA)-actuated exoskeleton used in active rehabilitation therapies for elbow joints. The sEMG signals are filtered and normalized according to data collected online during the first seconds of a therapy session. The control algorithm uses the sEMG signals to promote active participation of patients during the therapy session. In order to generate the reference position pattern with good precision, the sEMG normalized signal is compared with a pressure sensor signal to detect the intention of each movement. The algorithm was tested in simulations and with healthy people for control of an elbow exoskeleton in flexion–extension movements. The results indicate that sEMG signals from elbow muscles, in combination with pressure sensors that measure arm–exoskeleton interaction, can be used as inputs for the control algorithm, which adapts the reference for exoskeleton movements according to a patient’s intention.

## 1. Introduction

The development of advanced robotic assistive technologies has gained special attention in the scientific community over the last decades. Millions of people worldwide rely on assistive devices to improve their quality of life. For this reason, there is a need to further promote the development of assistive devices by pooling the efforts of engineers and clinicians, together with the feedback and experiences of users, to improve these technologies.

Aging of populations, mainly in developed countries, and the incidence of diseases, such as stroke, spinal cord injuries, and various musculoskeletal injuries, have increased the need for health resources, especially those dedicated to the rehabilitation process. Rehabilitation therapy is the process that assists a person in recovering from serious disorders after an injury, illness, or surgery that causes motor impairments. One of the most common rehabilitation methods consists of musculoskeletal rehabilitation to improve motor functions and the autonomy of patients in typical daily activities. In standard rehabilitation methods, every patient needs one or more therapists, because the therapist must directly manipulate the affected limb. This implies a huge consumption of healthcare and financial resources. The use of robotic devices as rehabilitation tools is proposed as a complement to the traditional rehabilitation sessions effectuated by therapists and can reduce the need for human resources. The main advantage offered by the use of robotic systems in rehabilitation is the capacity to support the work of physiotherapists in simple therapies with repetitive movements, reducing the need for the presence of the therapist. In this way, the costs associated with rehabilitation therapies can be reduced, allowing the same therapies to be carried out for longer, if the patient requires it, and for a larger number of patients to be treated simultaneously. Robotic systems have proven to be as effective as conventional therapy [1,2].

Among the most promising assistive robotic technologies are exoskeletons. An exoskeleton robot is a wearable robot designed to assist limb motions. The ease of use and the intuitive control of the robotic exoskeleton are crucial aspects for acceptance by patients. A step towards a more effective and intuitive control of upper-limb exoskeletons is the use of a myoelectric signal to detect the user’s motion intention. Myoelectric signals (MESs) contain information from which data about user movement intention in terms of muscular contractions can be extracted. Control based on MESs provides a more natural interaction with the exoskeleton.

A wearable shape memory alloy (SMA)-actuated exoskeleton with two degrees of freedom (DOF) (for flexion–extension and pronation–supination) was presented in [3]. In that work, the control algorithm made it possible to control the exoskeleton tracking a reference for passive rehabilitation therapy in flexion [4]—only actuating in flexion and recuperating (during the extension movement) with the aid of gravity—and actuating with two SMA-based actuators in flexion and extension [5]. The reference pattern in both cases represents a repetitive movement (for example, a sinusoidal trajectory) defined by the therapist, which makes the rehabilitation passive. In order to activate, in a natural manner, the exoskeleton according the user’s intended motion, the control algorithm proposed in this work uses input signals to the controller based on a skin surface electromyogram (sEMG). A key aspect for the success of robotic rehabilitation therapies is to keep the patient involved in carrying out the therapy. This is the objective pursued with the proposed control algorithm. Our new control algorithm analyzes the signal sEMG to detect that the patient is involved in the realization of the movement—that is, the patient intends to move their arm, even if they lack sufficient muscular strength to carry out the movement. The exoskeleton will only receive a reference position to which it will move if the patient is generating an sEMG signal indicating their intention to move.

In order to generate the reference position pattern with good precision, the sEMG normalized signal is compared with a pressure sensor signal to detect the intention to move. The pressure sensor is used to estimate the motion of the user through the force between the user and the robot. The proposed approach has been tested in a single joint for the flexion–extension task.

### 1.1. Electromyogram Signals

Electromyography (EMG) signals of human muscles are biological signals that record the electrical potential generated by muscle cells to contract. It can be used to detect the user’s intention to move, since the amplitude directly correlates with the user’s muscle activity. Moreover, according to [6], the EMG signal starts about 20–80 ms before the muscle contraction, so it allows anticipation of the motion intention.

EMG signals can be classified into two types: intramuscular EMG signals, detected from inside of the muscles; and surface EMG signals (sEMGs), detected from the skin surface. The intramuscular EMG signals give a better muscle activation pattern, but their use requires an invasive extraction procedure. Therefore, skin surface EMG signals are used as input for control robotic systems. Although the extraction of sEMG signals is relatively simple, the precise estimation of the motion is difficult because of the variability of EMG signals, which can be affected by multiple factors. EMG signals vary from one person to another, and even between two sessions with the same person making the same movement. In addition, each joint movement involves the activation of many muscles, and one muscle can be involved in various joint movements. Factors such as the changes in limb posture affect the relationship between the EMG signal level and motion estimation. The anatomy and physiological conditions of the user, including any diseases, injuries, fatigue, or pain, also modify EMG signals. Consequently, control strategies that employ sEMG signals require adjusting the controller to the particular user and, in many cases, calibrating the system during each session. Therefore, raw EMG signals are not suitable as input signals to a controller. Data must be filtered and normalized using the maximum voluntary contraction (MVC) level of the user [7].

In the case of an elbow exoskeleton, it must taken into account that the human elbow motion is activated by antagonistic pairs of muscles—biceps (agonist) and triceps (antagonist). According to [8], the biceps brachii, brachioradialis, and brachialis muscles are involved in elbow flexion. Biceps muscles are easily accessible from the skin surface. For this reason, the sEMG electrode circuit used in this work was situated over the bicep muscles to detect the intention of movement in the elbow joint.

### 1.2. Related Work

Since the 1960s, sEMG signals have been a common way of controlling prostheses [9,10]. More recently, EMG signals have been used for motion control of numerous robotic systems [11,12], prostheses [13], and robotics exoskeletons [14]. A broad review of the related literature can be found in [15].

Prosthesis and exoskeleton movements have frequently been controlled using EMG signals from muscles not involved in the movement. For example, Benjuya and Kenny [14] used the EMG signals from the wrist extensors of the forearm to open/close a pinch action. Also, in [7], the EMG signal from the ipsilateral biceps was used to develop an extremely reliable natural reaching and pinching algorithm. The EMG signals from the residual biceps and triceps of a user with transhumeral amputation have been proposed to control a robotic elbow in a learning from demonstration approach [16].

In the last decades, several research groups have worked on different control algorithms based on EMG signals for use with prostheses and exoskeletons. Many of these works have focused on the use of neural networks and fuzzy algorithms to distinguish the user’s intention for movement based on the EMG signals of various muscles. Hudgins [17] proved that artificial neural networks are practical for controlling prostheses by classifying different movements from EMG signals. In [18], the authors evaluated a time-delayed artificial neural network to predict shoulder and elbow motions using only EMG signals from six shoulder and elbow muscles as inputs. Results from both able-bodied subjects and subjects with tetraplegia indicate that the EMG signals contain a significant amount of information about arm movement that could be exploited in advanced control systems.

In [19], a hierarchical neurofuzzy controller based on the EMG signals was presented for real-time control of a shoulder and elbow motion exoskeleton. A wrist force sensor was used when the EMG activity levels were low. In [20,21], an EMG signal-based control method for a seven degrees of freedom (7DOF) upper-limb motion assistive exoskeleton robot (SUEFUL-7) was proposed. In their method, an impedance controller was applied to the muscle-model-oriented control method. Impedance parameters were adjusted in real-time as a function of the upper-limb posture and EMG activity levels. The work presented in [22] proposes a more advanced EMG-based impedance control method for an upper-limb exoskeleton. In that work, a neurofuzzy matrix modifier made the controller adaptable to all upper-limb postures of any user. The neurofuzzy modifier is a neural network with fuzzy reasoning that is trained to adjust its output to each user before operation. The method was applied to the 7DOF exoskeleton for upper-limb joint motions, as presented in [20]. They used 16 channels of EMG signals, with each electrode mainly corresponding to one muscle. Moreover, two force/torque sensors were used to estimate the forces between robot and user. The control algorithm was able to distinguish between different kinds of motion.

As can be seen from the previous studies cited, the EMG-based neurofuzzy control method has proven its effectiveness in controlling exoskeleton robots. However, the rules of control are complicated when increasing the number of degrees of freedom of the exoskeleton.

The amplitude of the EMG signals reflects the muscles’ activity levels. Many methods have been developed to estimate human muscular torque from EMG activity levels, using this information to control joint torques in robots. Due to the many factors that modify the EMG signals, this type of control requires a complex calibration process to adapt to the variability of the signals, and depends on the user and the session conditions. In the experimental work presented in [23], the reactions of 10 healthy subjects to the assistance provided through a proportional EMG control applied by an elbow powered exoskeleton were studied. The system did not require calibration. Their results showed that in order to assist movement, an accurate estimate of the muscular torque may be unnecessary and a simpler control algorithm can be more efficient.

The control algorithm presented in this work is similar to the binary control algorithm used in [7,24]. In [7], DiCicco tested binary “on–off” control, and variable and natural control algorithms based on EMG signals. They validated that the EMG signal from the ipsilateral biceps could be used to develop an extremely reliable natural reaching and pinching algorithm. A specific EMG threshold value serves to determinate the output binary value “on” if the EMG signal from the biceps muscle is above the threshold and “off” when it is below.

In our case, the rehabilitation exoskeleton has been designed with the objective of assisting in therapies consisting of performing repetitive movements. This type of therapy is typical of the first phases of rehabilitation, where the patient must repeat defined movements of a certain joint in order to recover muscular strength and increase the range of motion lost. In this context, it is not necessary to discriminate the type of movement that the patient wants to make. The proposed algorithm tries to determine the intention of the patient to initiate a certain movement and its ability to maintain it, even if the patient lacks sufficient muscular strength to carry it out. Consequently, the sEMG signals are detected and analyzed only from muscles directly related to the movement being assisted. In this case, the biceps muscles were targeted to detect voluntary flexion of the elbow joint. In the proposed algorithm, the triceps muscle activity was not considered, as the control algorithm has the limitation that if co-contraction happens and the extension signal is not detected by the pressure sensors, the system needs to be manually turned off.

Our proposed approach fuses sensor data with EMG signals. Force sensors were used to check the interaction between the exoskeleton and the user. In this way, only when the patient actively tries to execute the movement does the control algorithm initiate the movement of the exoskeleton. A similar approach was implemented in [20]. This approach reduces errors caused by low EMG levels or external unexpected forces affecting the patient’s arm.

This paper presents an algorithm capable of generating the reference pattern in position and torque based on surface electromyography (sEMG) signals and pressure sensors for high-level control of the SMA exoskeleton. The first part of the paper presents an introduction to the problem. In the second section, materials and methods are explained, including a description of the elbow exoskeleton. The initial assembly of SMA-based actuators is presented, and the elbow exoskeleton design is shown. The electronic hardware is also presented in the second section. The final part of the second section is devoted to explaining the high-level control algorithm in detail. In the third section, the results are presented: first, simulation test results of the high-level control algorithm, followed by performance evaluation of the proposed control method, based on experiments with healthy subjects that were carried out with the SMA elbow exoskeleton. The final part presents brief conclusions of the paper.

## 2. Materials and Methods

This section presents a brief description of the hardware architecture on which the tests were run: the structure of the exoskeleton, the actuators, and the sensors which are involved in the algorithm, as well as the high-level control algorithm capable of generating the reference patterns for position and torque; the algorithm provides high-level control and is based on sEMG signals and pressures sensors.

### 2.1. Elbow SMA Exoskeleton

In previous publications, a wearable SMA exoskeleton was presented with two DOF, which permits mobilization of the elbow joint in flexion–extension and pronation–supination movements [3,5]. This device used an SMA actuator for the actuation system and was the first elbow joint rehabilitation device powered by this technology. It has the potential to be a light device, with a weight less than 1 kg (structure, actuators, and electronics), noiseless operation, and low-cost fabrication. The actuator structure is described in Section 2.1.1.

#### 2.1.1. Actuator Design

**Figure 1:**
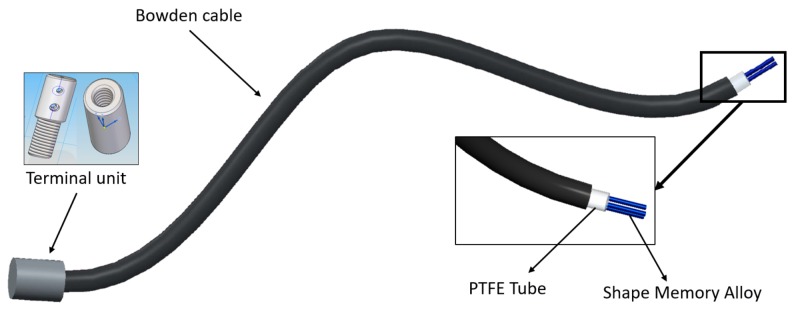
Actuator design. Flexible shape memory alloy (SMA)-based actuator.

The simple SMA-based actuator (with only one SMA wire) used in this work was presented in [25]. The SMA wire is made of a metallic alloy—a common mixture of nickel and titanium, called Nitinol [26]. It has the property of recovering its original shape (memorized shape) between two thermic transformation phases: the martensite phase (at low temperature) and austenite phase (at high temperature). The principle on which it works is based on the heating effect (Joule effect), where electrical energy is transformed into thermal energy, after which the thermal energy is transformed into mechanical energy. During this transformation, the SMA wire undergoes a variation of total length, between 3% and 5%. As a function of the diameter and alloy type, the actuator can exert different forces. A 0.51 mm diameter wire of Flexinol^®^ [26] can exert a force of about 35.6 N (with a lifetime of tens of millions of cycles under these force conditions). The SmartFlex^®^ [27] wire with the same diameter can exert a maximum force of 118 N (with a lifetime of hundreds or a few thousand cycles). The activation temperature of the SMA wire depends on the alloy and, in this case, it is 90 °C. In this work, the actuator was composed of multiple SMA wires, a polytetrafluoroethylene (PTFE) tube, a Bowden tube, and the terminal parts (Figure 1).

- The Bowden cable is a mechanical flexible cable which consists of a flexible inner cable that forms a metal spiral and a flexible outer nylon sheath. This type of wire can guide the SMA actuators and transmit the force. In addition, the metal has the property of dissipating the heat, which is an advantage during the recuperation of the initial position phase.
- The PTFE tube can support high temperatures, more than 250 °C; it is an electrical insulator and does not cause friction.
- The terminal units are used at one end to connect the actuator to the actuated system and at the other to fix the SMA wires to the Bowden cable. They also serve as connectors for the power supply (using the control signal). These units are formed of two pieces that can be screwed to each other to set the tension of the SMA wires. The total SMA wire tension range adjustment is 0.01 m.

There is a relation between the SMA wire diameter, the force, and the cooling time (Table 1). In Table 1, the first column represents the diameter of the wire, the second column is the actuation force which guarantees a lifetime of tens of millions of cycles, and the last two columns represent the cooling time for the two types of wires, with activation at 70 °C and 90 °C, respectively. According to the data shown in the table and the objectives of the exoskeleton, it was decided to work with 0.51 mm wires activated at 90 °C, because the maximum force was obtained with this diameter and the cooling time is lower than when the wire was activated at 70 °C.

If the SMA actuator is designed to operate with the configuration parameters shown in Table 1, the actuator lifetime can be tens of millions of cycles. If the actuator operates with higher forces than those specified, the lifetime drops to only a few thousand cycles.

Regarding the application of the necessary torque to execute defined movements (the necessary torque of each movement was found from a biomechanical simulation [3]), a summary of the system configuration of the actuators can be seen in the Table 2.

#### 2.1.2. Exoskeleton Design

**Figure 2:**
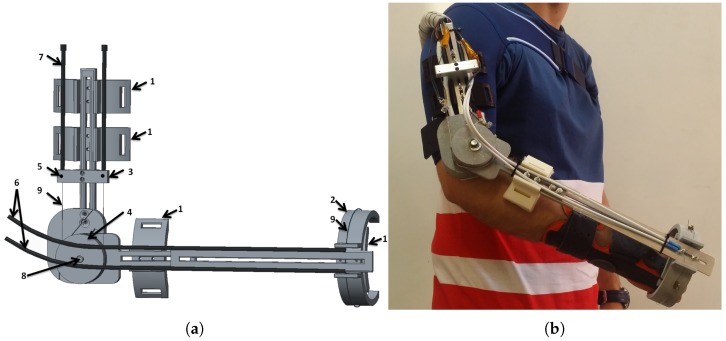
SMA exoskeleton design. (**a**) CAD structure: 1—attachment points with the hand and force-sensing resistor (FSR) sensors, 2—fixed structure for supination–pronation, 3—actuator termination for Bowden tube, 4—pulley for linear to rotational transformation, 5—temperature sensors, 6—supination–pronation actuators, 7—flexion–extension actuators, 8—absolute encoder, 9—SMA wires. (**b**) SMA elbow joint exoskeleton on a human body.

The exoskeleton was designed according to elbow biomechanics. A biomechanical simulation was performed with the objective of finding the necessary force for various frequencies of movement [3] using the actuator structure presented in Section 2.1.1. The structure of the exoskeleton is displayed in the Figure 2. It was made using simple parts that can be assembled easily, and it permits matching the dimensions of the exoskeleton to those of the user (length of the arm and the forearm), such that the axis of the elbow joint remains aligned with the axis of the exoskeleton. The components of the exoskeleton were a combination of aluminum pieces (such as the Bowden terminals and axis) and other parts made by 3D printer using aluminum with polyamide. The exoskeleton has four points of attachment to the human body, connecting with the arm (two attachments), the forearm, and the hand (Figure 2a). Three force-sensing resistors (FSRs) were placed in the hand piece. These can measure a force between 0.1 and 10 kg. For the safety of the patient, the exoskeleton movement is mechanically limited between 0 and 150 degrees in the elbow flexion–extension direction and between 70 and −70 degrees in the supination–pronation direction. In order to increase comfort, all internal parts in contact with the patient were covered with a soft hypoallergenic material. Compared with current solutions, due to the lack of gears and motors in the mechanism, the proposed rehabilitation device is lightweight. The whole structure with the actuators weighed less than 1 kg. A 960 W DIN rail power supply (24 Vdc/40 A) was used to provide the necessary energy to the actuators. The weight of the power supply unit was 1.9 kg. In addition, it provides noiseless operation, which increases the comfort of the patient during the rehabilitation process. The final version of the exoskeleton installed on the human body can be seen in Figure 2b.

#### 2.1.3. Electronic Hardware

The electronic hardware is composed of power electronics, a controller, and sensors placed in the device. The power electronics are capable of supplying the necessary power to four distinct actuators: flexion, extension, supination, and pronation. The system is based on a MOSFET transistor (STMicroelectronics STP310N10F7, STMicroelectronics group, Shanghai, China), which works as a switch circuit and amplifies the control signal (PWM) generated by the controller. The device was connected to the terminal units of the SMA-based actuator.

The controller is a 32-bit microcontroller STM32F4 from STMicroelectronics ^®^, China, which can be fully programmed with Matlab/Simulink^®^ [28]. It was programmed with four different PWM output ports, which generate the necessary duty cycle for managing the four actuators (each with one or more SMA wires).

The structure of the rehabilitation device includes sensors for position, temperature, force, and sEMG. An absolute angle position sensor with Hall effect (AS5045 made by AMS (Austrian Micro Systems), Premstaetten, Austria) is placed in the shaft of the exoskeleton (pulley for flexion–extension). This sensor has a resolution of 0.0879 degrees and measures the flexion–extension movement. The second position sensor, a membrane potentiometer made by Spectrasymbol, has a length of 0.1 m and is placed on the supination–pronation piece (on the outside) to measure the absolute displacement of this movement. In the same piece, on the inner part which makes the connection between the human forearm and hand and the exoskeleton, three FSR sensors were placed with a 60-degree angular distance between them. These sensors measure the force variation of the elbow during flexion–extension movements—forces that are involved in the high-level control algorithm. Another main sensor involved in this algorithm is the sEMG sensor. The circuit uses three disposable disc electrodes, F-TC1 made by SKINTACT—a low-cost, multi-purpose ECG. It consists of Ag/AgCl electrodes, a conductive gel (Aqua-Tac), an adhesive area with a dimension of 35 × 41 mm, and a snap connection. The gel permits a better connection between the skin and the electrode. This electrode is in the category of non-invasive and wet electrodes.

**Figure 3:**
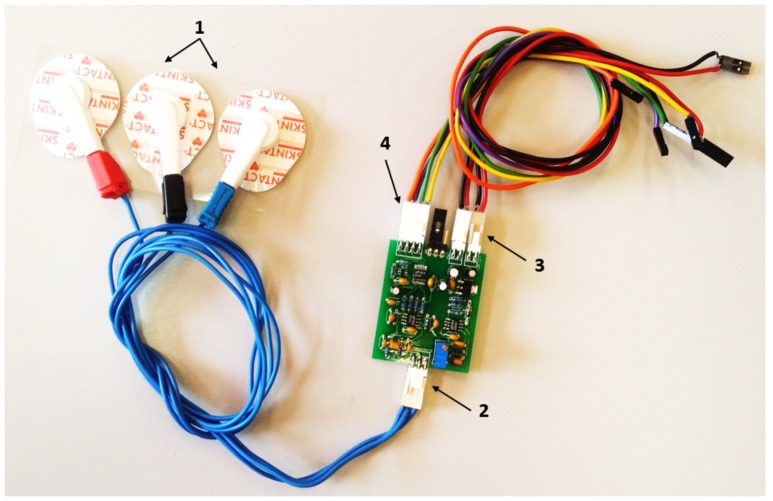
Surface electromyography (sEMG) circuit with two channels and the electrodes: 1—electrodes, 2—electrode connector, 3—connectors for power supply (5 V and GND), 4—connector for serial peripheral interface (SPI) communication.

The sEMG circuit (Figure 3) was made in Carlos III University of Madrid (UC3M), and presents two channels that are connected by two electrodes, which are situated at a distance of 0.03 m from each other over the belly biceps muscle; another channel is used as a reference, which is connected to the last electrode positioned over the shoulder blade. The EMG circuit is composed of various stages, including connectors. There is the differential active feedback stage, the digital stage (where the signal is amplified and filtered), and the stage for the power supply and communication connectors. The communication between the EMG and the microcontroller uses a serial peripheral interface (SPI) bus. For the signal-processing module, we used the same microcontroller STM32F4.

The temperature sensors are placed in the terminal of the actuator to measure the temperature of the SMA wires, a parameter that is required in the control loop. All the electronics used in this project were based on low-cost components.

**Figure 4:**
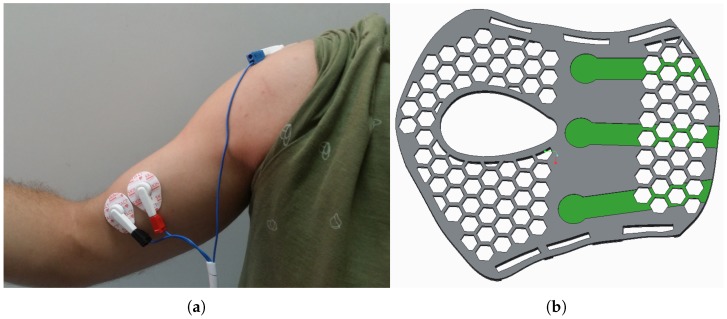
(**a**) Surface electromyography (sEMG) electrodes over the subject’s arm and shoulder blade. (**b**) The auxiliary piece where are placed the FSR sensors (green parts).

The position of the EMG electrodes and FSR sensors over the human body can been seen in Figure 4. A auxiliary piece was built to form the connection between the human hand and forearm-sensor-exoskeleton. This piece (made with a 3D printer using PLA (polylactic acid)) was bent (by introducing it to hot water before the sensors were mounted), taking the form of the patient’s forearm, and formed the connection between the forearm and hand with the exoskeleton.

### 2.2. The High-Level Control Algorithm

Previous publications [3,5] presented a low-level control algorithm based on a BPID (bilinear proportional integral derivative) controller, which governs the SMA-based exoskeleton in position. Their algorithm, involving position and temperature sensors, is capable of acquiring data from the sensors or controlling the exoskeleton in flexion, extension, or in flexion–extension using an antagonistic controller (two BPID controllers in a parallel configuration [5]). With the data acquisition configuration, the SMA-based exoskeleton only offers the possibility to diagnose and evaluate the patient. In the passive mode, the actuators offer all the necessary force to reach and follow the reference position without taking into account the patient force. Through the introduction of sensors for pressure/force and sEMG, the SMA-based exoskeleton offers the possibility of rehabilitation therapies in active mode, where the reference position is generated by the patient’s movement intention. In this way, passive reference position (habitually sinusoidal movements) is changed to active reference in a case where the patient presents activity in the motor function (the motor function has been partially affected). Active reference involves the patient undergoing rehabilitation therapy, leading to a faster recovery. The high-level control algorithm, which generates the active rehabilitation therapy (active reference position), uses the sEMG sensors and force-sensing resistor (FSR) sensors, together with position sensors. This is currently available (due to the SMA-based exoskeleton configuration—in fact, the sensors) only for the elbow flexion movement.

The sEMG signals are captured at a sampling frequency of 1 kHz using the circuit presented in Section 2.1.3. The signals are preprocessed: firstly, the raw sEMG data is filtered with a band-pass Butterworth filter, order 8, with a cut-off frequency at 6 dB point below the band-pass value of 20 Hz, and the second cut-off frequency with a value of 480 Hz. This filter was proposed in order to remove the movement artifact [29]. After that, the absolute value of the response of the filter is calculated, and this value is provided to the second filter. This is a low-pass Butterworth filter, order 10, with a cut-off frequency of 20 Hz. The 20 Hz cut-off frequency of the low-pass filter was decided upon according to [29], wherein the authors claim that in the last three decades, various recommendations and standards have been put forth for a cut-off frequency between 5 and 20 Hz. In his publication, he chose such an adequate cut-off frequency of 20 Hz. Both filters were configured at a frequency of 1 kHz. After the filtering process, the EMG signal proceeds to the normalization stage. This consists of an online calibration, where the first 2 s are ignored (in the first 2 s, the circuit experiences some perturbation), and the next 18 s are used to detect the maximum and minimum signals for the normalization process. In this time, the patient is required to flex the forearm as much as possible at least once, followed by an extension movement to return to the original position. During these 18 s, maximum and minimum values are stored to be used in the normalization process, where the normalized signal, $E_{norm}$, is calculated by Equation (1):

(1) $$E_{norm}=\frac{E_{act}-E_{min}}{E_{max}-E_{min}};$$

where $E_{act}$ is the actual EMG signal, and $E_{min}$ and $E_{max}$ are the minimum and maximum values of the EMG signal during the 18 s used for normalization.

**Figure 5:**
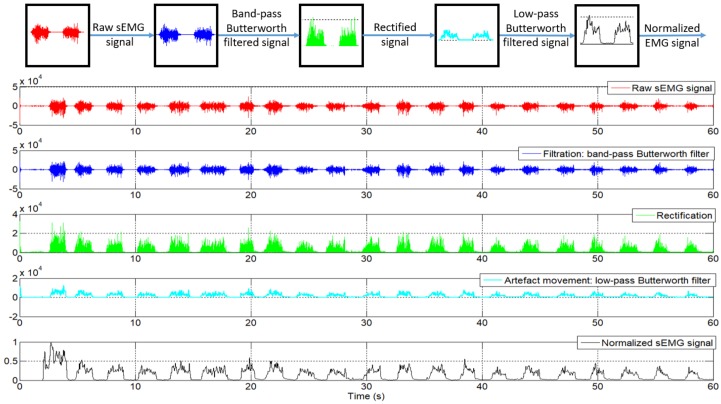
Surface electromyography (sEMG) signals after each processed step.

The entire process of filtering and normalizing of the sEMG signals can be seen in Figure 5.

**Figure 6:**
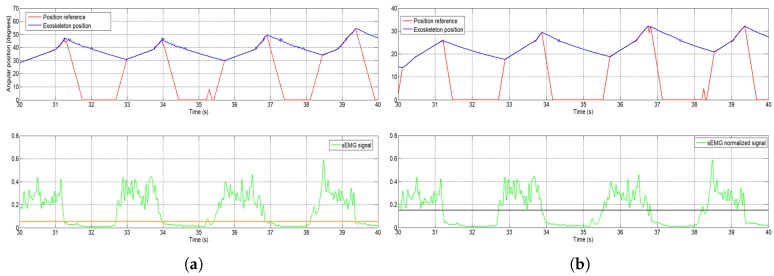
(**a**) the orange line shows data using a 0.05 threshold. (**b**) the black line shows data using a 0.15 threshold.

The normalized signal is compared with a threshold value between 0 and 1. This threshold value is fixed experimentally according to the patient and the desired sensitivity of the algorithm. Lower threshold values imply that the algorithm will be more sensitive to the EMG signal and detect motion intention with less signal intensity, but may be more affected by unexpected external forces. The effect of the threshold, using the same sEMG signals with different thresholds, can be seen in Figure 6. The result of this comparison represents the intention of movement detected through the sEMG signal from the biceps muscle—more precisely, the elbow flexion.

If the sEMG signal decreases over time, then the following should be taken into account. If $E_{act}$ becomes less than $E_{min}$, the result according to Equation (1) is a negative signal, which causes the actuators to turn off (the intention of movement is not detected). If the patient’s intent is to move the forearm and the result of Equation (1) is less than the threshold, the value of this can be modified online to change the sensibility of the algorithm. If $E_{act}$ becomes less than $E_{min}$ when the patient’s intent is to move the forearm, the algorithm needs to be recalibrated.

The proposed control algorithm generates the reference position as an increment of the current joint angle. That is, if movement intention is detected in the sEMG signal, the control algorithm provides a reference to increase the elbow angle of flexion. If no movement intention is detected, the reference position will be null and the actuator is disabled.

According to the actual elbow position and the final movement intention, the system works between two types of increments: one for fast reference position generation and another used to generate a slow reference position. The first increment is used when the actual position of the elbow joint is different from the position of the actuator reference. This case occurs when motion intention is detected, that is, the signal sEMG exceeds the established threshold after a period of deactivation of the actuators caused by the non-detection of intention to move. The exoskeleton used in the flexion movement leaves the joint free to move, as long as the actuator is not activated because of the loss of patient motivation and engagement that results in loss of the EMG signal. At that moment, the reference position is zero, but the actual joint position is not null. This situation is shown in the descending part of the sawtooth-shaped graph in Figure 6. The loss of intention to move produces a null reference that causes deactivation of the actuator, and the recovery of the intention causes a rapid increase of the reference position. If the algorithm is activated and detects an intention to move, the generated reference uses a fast increment until it reaches the elbow position, after which it uses a slow increment to generate the reference that will be followed by the exoskeleton, as long as there exists an intention to move. When intention to move is no longer detected, the high increment is used to decrease the reference position; the actuators are no longer activated and the extension movement is carried out by actuator recuperation (dissipation of the heat).

In order to address the situation caused by small EMG levels and generate the reference position pattern with better precision, the high-level control algorithm uses the sEMG normalized signal together with the FSR sensor signal. Similar to the EMG signals, the signal from the FSR sensors is filtered and normalized. The filter for this signal is a low-pass filter at a frequency of 100 Hz. Filtered signals are normalized in the same way as the sEMG signal, using an equation analogous to (1). After that, it is compared with the threshold defined to detect the intention to move through the force interaction between the patient and exoskeleton. For flexion movement detection, only the signal provided by the FSR sensor placed over the radius bone is taken into account. The patient’s movement intention causes the forearm to exert pressure over the rigid part of the exoskeleton, which can be detected with this sensor. The two signals, from sEMG and FSR, are logically compared in order to detect the final intention to move, a binary result that is used later. The logical comparison consists of an AND function, to ensure a higher accuracy of the algorithm, having a minimum of two active signals (above the threshold), or with an OR condition if the reference is generated, where at least one of the signals is above the threshold.

**Figure 7:**
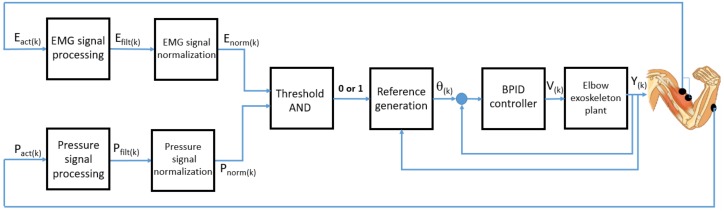
High-level control algorithm based on EMG and pressure signals for reference position generation.

The scheme of the high-level control algorithm capable of generating the reference position pattern can be seen in Figure 7, where $E_{act(k)}$ and $P_{act(k)}$ are the actual EMG and pressure or force signals in the discrete domain, $E_{filt(k)}$ and $P_{filt(k)}$ are filtered EMG and pressure or force signals, $E_{norm(k)}$ and $P_{norm(k)}$ are normalized EMG and pressure or force signals, $\theta_{\left(k\right)}$ is the generated angle reference, $V_{\left(k\right)}$ is the control signal, and $Y_{\left(k\right)}$ is the angular position of the SMA-based exoskeleton.

In parallel to the algorithm that generates the reference position, the normalized EMG signal is used to generate a torque assistive reference for rehabilitation therapy. According to the total height and weight of the patient, the weight of the forearm and hand is approximately calculated, as well as the length from the joints to the center of gravity of each. As a function of these parameters and the actual angle, torque on the elbow joint is estimated. Using this torque and the sEMG signal, a percentage of assistance in torque reference can be generated. This percentage can be set by the user. Torque assistive reference is directly proportional to the sEMG signal. A similar idea is presented in [30], but they did not take the biomechanical structure of the human body into account.

## 3. Results

In order to highlight the algorithm performance, feasibility, and adaptability to various hardware configurations, a series of tests were done. Firstly, simulation with EMG signals from different circuits, together with an actuator model, was conducted to simulate the behavior of the actuator in the exoskeleton; secondly, the real hardware over the exoskeleton was tested with healthy subjects.

### 3.1. Results of Simulation

In [31], the model of an SMA-based actuator with a variable charge was presented. This permits the simulation of the actuator with different SMA diameters (0.51 mm and 0.1 mm); in this case, the 0.51 mm diameter was used. According to the simulation results presented in [31], which were compared with the real behavior of an SMA actuator, it can be concluded that the behavior of the model is highly similar to a real actuator. To use this model in the simulation with the high-level control algorithm based on sEMG, a number of settings of the SMA-based actuator were used. Firstly, the charge of the actuator was set according to the forearm and hand weight, and the linear position was converted to an angular position as a function of the exoskeleton characteristics, such as the pulley radius. It is worth noting that the SMA-based actuator model includes the same low-level control algorithm ([3,5]), as well as the exoskeleton.

For the sEMG data acquisition, the electrodes were placed along the biceps muscle fibers and on the midline of the belly of the muscle, taking into consideration that this is where the sEMG signals have the greatest amplitude (Figure 4). The subject was asked to perform some elbow extension–flexion movements, and data was saved to be used in offline simulation. This process was accomplished with two types of sEMG circuits: firstly with the circuit realized in UC3M, presented in Section 2.1.3, and secondly with an sEMG measurement device (DKH Co., Ltd., Tokyo, Japan) with a sampling frequency of 1 kHz. This latter circuit was successfully used in other works, such as for the control of a prosthetic hand [32], and in a rehabilitation finger system [33]. The sEMG signal acquired with the UC3M circuit was similar to the sEMG signal acquired with the DKH circuit.

**Figure 8:**
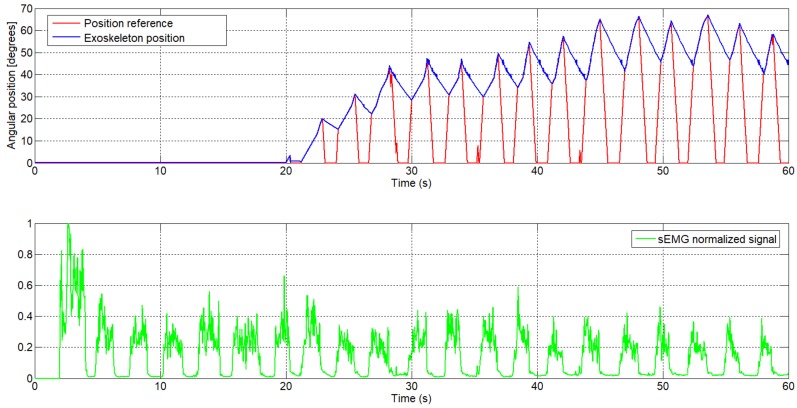
The generated angular reference position from the sEMG signal with the UC3M circuit: first subject (male, 24 years old, 1.73 m height, and 70 kg weight).

Figure 8 shows the normalized sEMG signal acquired from the UC3M circuit, the generated reference, and the angular position of the exoskeleton. This first test was realized offline in a simulation, where the signal of FSR sensor was set to 1 (this means that the signal of the FSR sensor is ignored), and the increment was set empirically to 0.1 for fast increment and 0.01 for slowly increment.

As can be seen, at t=20 s, the reference position is 0 degrees, since this signal from the sEMG was used for calibration, whereas the first 2 s were ignored for perturbation. Next, t=18 s were used to detect the maximum and minimum sEMG signal. After this process of calibration, starting at t=20 s, once muscle activity has been detected in the biceps muscle, the algorithm starts to generate the reference.

**Figure 9:**
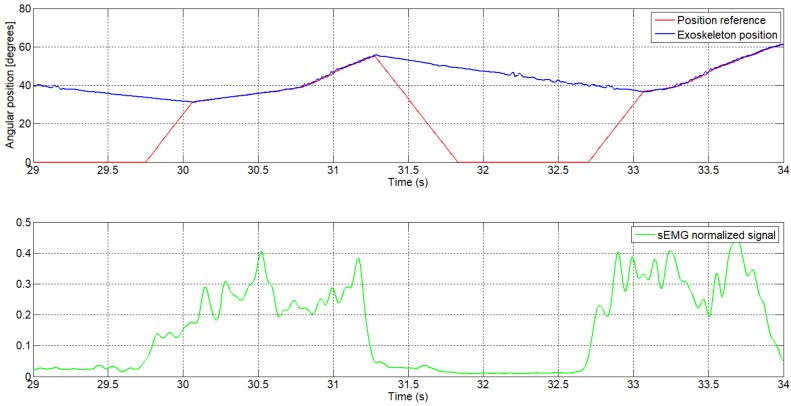
The angular reference position generated by the sEMG signal, first subject (enlarged area).

We take, as an example, the sEMG signal at t=29 s (Figure 9). From this moment, the normalized sEMG signal changes the amplitude, which means that the circuit detects muscular activity in the bicep muscles, and the algorithm begins to increment the reference position. Because the actual angular position of the exoskeleton is different to the actual reference, by approximately 30 degrees, the algorithm increases the angular reference position with a high increment. Once the angular reference position coincides with the exoskeleton position, the algorithm increases the angular reference position with a slow increment and the exoskeleton begins to follow the voluntary movement intention. In t=32.5 s, the amplitude of the normalized sEMG signal decreases, the high-level control algorithm interprets that there is no intention to move by the user and, therefore, the algorithm decreases the angular reference position. In this case, though the reference decreases very quickly, the angular position of the actuator is limited by the actuator behavior (shows a slow recovery due to heat accumulation). The sEMG threshold can easily be set from the user interface and, in this case, it was set to 0.05.

**Figure 10:**
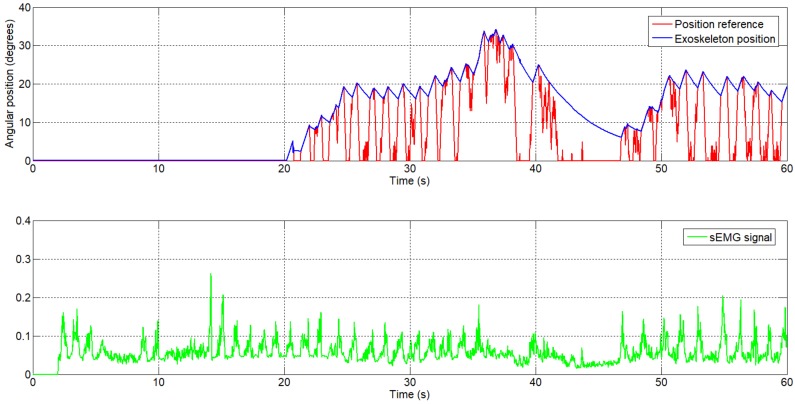
The generated angular reference position by the sEMG signal, second subject.

The second test was performed with a different sEMG circuit and a different person. Similar to the first case, the person was asked to execute some repetitions of flexion–extension of the elbow and the sEMG signal was recorded. The signal can be seen in Figure 10, from which can be observed a higher frequency of movement of the elbow joint. Between t=40 s and t=45 s, we can see a muscle relaxation; the amplitude of the sEMG signal decreases, and in this case the angular reference position goes to 0 degrees. The exoskeleton behavior can be seen when the extension actuator is not active: it represents a slow extension movement.

**Figure 11:**
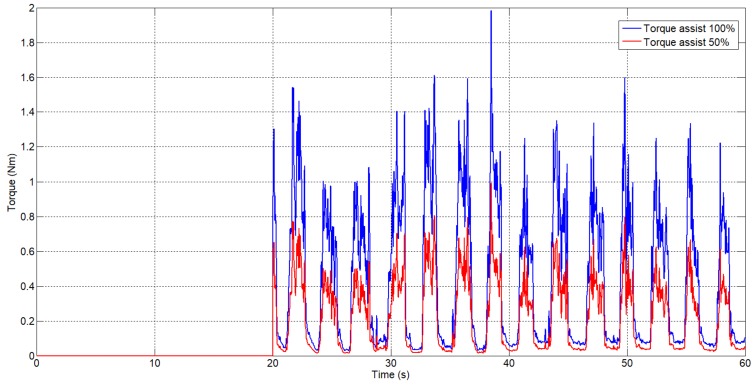
The generated torque reference from the sEMG signal.

In parallel, the algorithm offers the possibility to generate the torque reference to assist the movement. This reference is generated according to the biomechanical model of the human body, taking into account that rehabilitation is executed standing or sitting, and that the sEMG signal is detected over the biceps muscles. In Figure 11, the pattern reference in torque assistance is presented for one patient with a weight of 70 kg and height of 1.73 m for two cases: the exoskeleton assists the patient with the total torque, 100% (blue signal), and the exoskeleton assists with 50% of total torque (red signal). The sEMG signal used to generate this reference in torque assistance is the same as the sEMG signal presented in Figure 8.

### 3.2. Results with the Real SMA Exoskeleton

**Figure 12:**
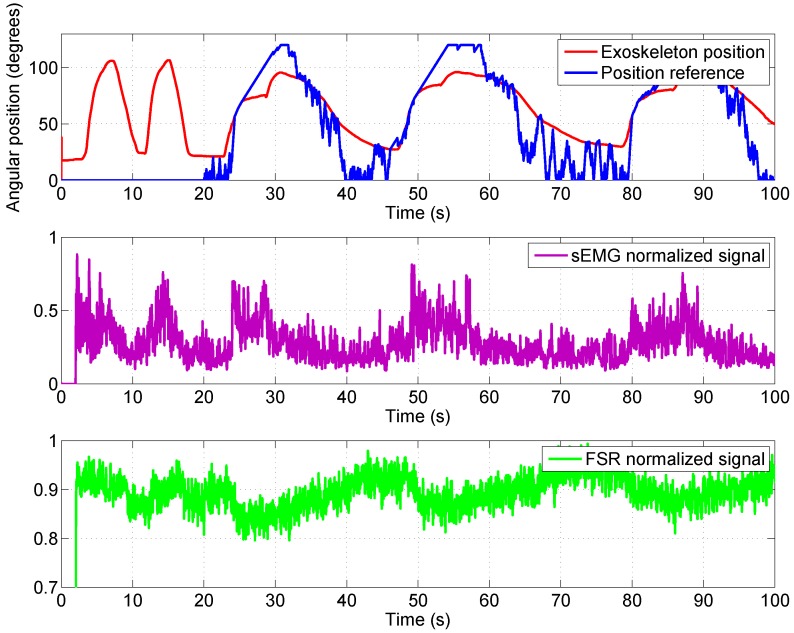
Reference position and response generated by the sEMG signal.

The sEMG-based control algorithm was tested in the real exoskeleton presented in Section 2.1. This was tested with healthy people from RoboticsLab laboratory, Carlos III University of Madrid. The characteristics of the subject were: male, 1.73 m height, and 70 kg weight. Firstly, sEMG electrodes were fixed over the biceps (over the belly of the biceps, a good positioning is essential) and the shoulder (reference electrode), and then the exoskeleton was fitted over the body. The exoskeleton was configured on the subject’s body so that the elbow axis was aligned with the exoskeleton rotation axis and the FSR sensors were in contact with the hand and forearm. The results of this test can be seen in Figure 12, showing the reference position signal (the blue signal), which is generated by the sEMG signal (purple) and the FSR signal (green), and the real position exoskeleton (red).

According to the high-level control algorithm, in the first 20 s, the exoskeleton user calibrates the algorithm through movements of flexion–extension of the elbow joint. In Figure 12, two movements of flexion–extension can be observed during the first 20 s. In these first seconds, the output reference is 0 degrees.

In the second graphic, the sEMG signals can be seen, where the amplitude is changing during the flexion–extension movement. In the third graphic is the FSR sensor signal variation corresponding to the flexion–extension movement. After the process of calibration, when the algorithm detects the movement intention (from the sEMG signal and FSR sensor), it starts to generate the reference position and the exoskeleton begins to move following the reference. We take as a reference example the interval t=23–40 s. At t=23 s, the FSR sensor presents a signal with a high amplitude which exceeds the value of the threshold, and the sEMG signal also begins to increase in amplitude. Starting from this point, the algorithm begins to generate the angular reference, incrementing slowly, as the angular reference is near the exoskeleton elbow position. Until t=30 s, the amplitude of the sEMG signal remains high, with the angular reference reaching the maximum 120 degrees. Due to the elbow movement, the FSR sensor signal amplitude may have decreased and, for this reason, the weight of this signal (during this period) on the algorithm is lower. After time t=30 s to t=40 s, the sEMG signal has decreased its amplitude and the algorithm starts to decrease the angular reference, finally to 0 degrees.

To successfully use the exoskeleton in this mode of rehabilitation therapy (active mode), the patient needs to present a minimum level of activity in the motor function, otherwise the algorithm is not capable of detecting the movement intention based on the sEMG and force/pressure signals. If this mode of therapy cannot be used by the patient, passive mode rehabilitation therapy can be used, where the exoskeleton follows a passive reference (habitually a sinusoidal reference).

## 4. Conclusions

In this work, a new high-level control algorithm based on sEMG signals and pressure/force signals capable of generating the angular and torque reference for an active rehabilitation was presented. An algorithm capable of generating the angular and torque reference was successfully tested: in simulations (with the EMG signals provided by the circuit made by the research group and a commercial circuit) and in real applications over the SMA elbow exoskeleton with healthy people. In the latter case, in a real device, the sEMG signal was used together with the force/pressure signals from an FSR sensor.

The SMA-based exoskeleton for an elbow joint presented in this work, together with the low and high-level control algorithm and sensors, is based on low-cost components and offers three modes of operation:

- Data acquisition mode: to evaluate and diagnose the patient. Also, in this mode of operation, the angular limits of elbow movement are saved to set the angular reference limits for the control algorithm.
- Passive rehabilitation mode: The exoskeleton follows a defined angular reference, the most common being a sinusoidal type. In this case, the patient executes repetitive movements, not taking into account the movement intention of the patient. The exoskeleton can support all the movement in flexion, extension, or flexion–extension.
- Active rehabilitation mode: The angular reference for the elbow exoskeleton is generated as a function of the patient’s intention for movement, detected by the sEMG signals and force/pressure signals. In this case, the patient is actively involved in the rehabilitation therapy, and if movement intention is not detected, the angular reference goes to 0 degrees. This type of rehabilitation can only be used with patients who present a minimum activity level in their motor function; otherwise, a passive rehabilitation can be used.

The performance of the high-level control algorithm considered the biceps muscle activity and did not take into account the triceps muscle activity. If this co-contraction appears, and the FSR sensor is not capable of detecting it, the user needs to manually stop the system.

The control algorithm presented in this paper permits the adjustment of the parameters of the generated reference position, such as the increments (which modify the angular velocity response of the exoskeleton) and the thresholds of the sEMG and FSR signals, which change the sensibility of the algorithm (from which signal value the algorithm starts to increment to the reference position).

The main advantage provided by the proposed high-level controller is that it forces the patient to be involved in the therapy task on a constant basis. If the patient loses attention, the exoskeleton is deactivated. In this way, the controller promotes active rehabilitation.

## Figures and Tables

**Table 1:** SMA wire characteristics [26].

Diameter Size [mm]	Force [N]	Cooling Time 70 °C [s]	Cooling Time 90 °C [s]
0.025	0.0089	0.18	0.15
0.038	0.02	0.24	0.2
0.050	0.36	0.4	0.3
0.076	0.80	0.8	0.7
0.100	1.43	1.1	0.9
0.130	2.23	1.6	1.4
0.150	3.21	2.0	1.7
0.200	5.70	3.2	2.7
0.250	8.91	5.4	4.5
0.310	12.80	8.1	6.8
0.380	22.50	10.5	8.8
0.510	35.60	16.8	14.0

**Table 2:** Exoskeleton actuators.

Movement	SMA Wires	Maximum Actuator Force [N]	Length [m]	Weight [kg]
Flexion	3	354	1.5	0.16
Extension	2	236	1.5	0.15
Pronation	1	118	2	0.1
Supination	1	118	2	0.1

## Abbreviations

The following abbreviations are used in this manuscript:

SMA	Shape Memory Alloy
UC3M	Carlos III University of Madrid
FSR	Force Sensing Resistor
PWM	Pulse-Width Modulation
sEMG	Surface electromyography
PTFE	Polytetrafluoroethylene
DOF	Degrees of freedom
SPI	Serial Peripheral Interface
PLA	Polylactic Acid
MES	Myoelectric signals
